# Is the exquisite specificity of lymphocytes generated by thymic selection or due to evolution?

**DOI:** 10.3389/fimmu.2024.1266349

**Published:** 2024-03-25

**Authors:** Rob J. De Boer, Can Kesmir, Alan S. Perelson, José A. M. Borghans

**Affiliations:** ^1^ Theoretical Biology and Bioinformatics, Utrecht University, Utrecht, Netherlands; ^2^ Department of Theoretical Biology and Biophysics, Los Alamos National Laboratory, Los Alamos, NM, United States; ^3^ Center for Translational Immunology, University Medical Center Utrecht, Utrecht, Netherlands

**Keywords:** T cell specificity, T cell repertoire, negative selection, evolution, repertoire diversity

## Abstract

We have previously argued that the antigen receptors of T and B lymphocytes evolved to be sufficiently specific to avoid massive deletion of clonotypes by negative selection. Their optimal ‘specificity’ level, i.e., probability of binding any particular epitope, was shown to be inversely related to the number of self-antigens that the cells have to be tolerant to. Experiments have demonstrated that T lymphocytes also *become* more specific during negative selection in the thymus, because cells expressing the most crossreactive receptors have the highest likelihood of binding a self-antigen, and hence to be tolerized (i.e., deleted, anergized, or diverted into a regulatory T cell phenotype). Thus, there are two —not mutually exclusive— explanations for the exquisite specificity of T cells, one involving evolution and the other thymic selection. To better understand the impact of both, we extend a previously developed mathematical model by allowing for T cells with very different binding probabilities in the pre-selection repertoire. We confirm that negative selection tends to tolerize the most crossreactive clonotypes. As a result, the average level of specificity in the functional *post-selection* repertoire depends on the number of self-antigens, even if there is no evolutionary optimization of binding probabilities. However, the evolutionary optimal range of binding probabilities in the *pre-selection* repertoire also depends on the number of self-antigens. Species with more self antigens need more specific pre-selection repertoires to avoid excessive loss of T cells during thymic selection, and hence mount protective immune responses. We conclude that both evolution and negative selection are responsible for the high level of specificity of lymphocytes.

## 1 Introduction

The repertoires of B- and T-lymphocytes in the adaptive immune system are extremely diverse. The diversity of T-cell receptors (TCRs) in the circulating pools of naive CD4^+^ and CD8^+^ T cells in human adults has been estimated to be more than 10^9^ unique *αβ*-TCRs (1). Repertoires need to be diverse because the antigen receptors expressed by lymphocytes are very specific. For instance, the precursor frequency for a typical viral epitope is about one cell in 10^5^ to 10^6^ naive CD8^+^ T cells (2–7). A repertoire therefore needs to contain many more than 10^5^ unique antigen receptors to be complete, i.e., to be expected to mount an immune response to any foreign antigen (8, 9). To avoid autoimmunity, lymphocyte receptors binding self-antigens should be absent from the circulating repertoire of functional naive T cells (or have adopted an unresponsive phenotype). For the peptides of nine amino acids (9-mers) that are used as epitopes by CD8^+^ T cells, we estimated that there are about 10^7^ unique self-epitopes in the human proteome, of which about 10^5^ are expected to be presentable on a particular HLA molecule (10) as a unique peptide-MHC (pMHC). Thus, any naive CD8^+^ T cell faces the problem of having to respond to about one in 10^5^ to 10^6^ foreign pMHCs, while not binding any of the about 10^5^ self-pMHC presented on the MHC molecules it is restricted to.

Although low precursor frequencies confirm that lymphocytes tend to be very specific, i.e., have a low probability to bind a randomly chosen antigen, it is also well-known that a typical TCR can bind many different peptides, which even do not need to be similar (11). Wooldridge et al. showed that one particular TCR (i.e., 1E6 binding an A*0201-restricted 10-mer) was able to bind over a million different peptides with sufficient affinity (12). TCRs are therefore said to be broadly specific, cross-reactive, degenerate, and promiscuous (13). Instead, the ‘exquisite specificity’ that we are studying here is defined as the probability a TCR binds a randomly chosen pMHC. Thus, highly-specific TCRs have a low binding probability, *p*. Most authors agree that a high level of TCR specificity is perfectly compatible with the ability of a TCR to bind many pMHC, simply because there are so many different pMHC (12–14). For example, there are $20^{10}≃10^{13}$ different 10-mers, and binding more than 10^6^ of them (12), would still be compatible with a low binding probability of *p <* 10^−6^, which is a normal precursor frequency. Since TCRs may differ widely in their levels of specificity, we extend previous models that were based upon a single binding probability.

**Figure 1:**
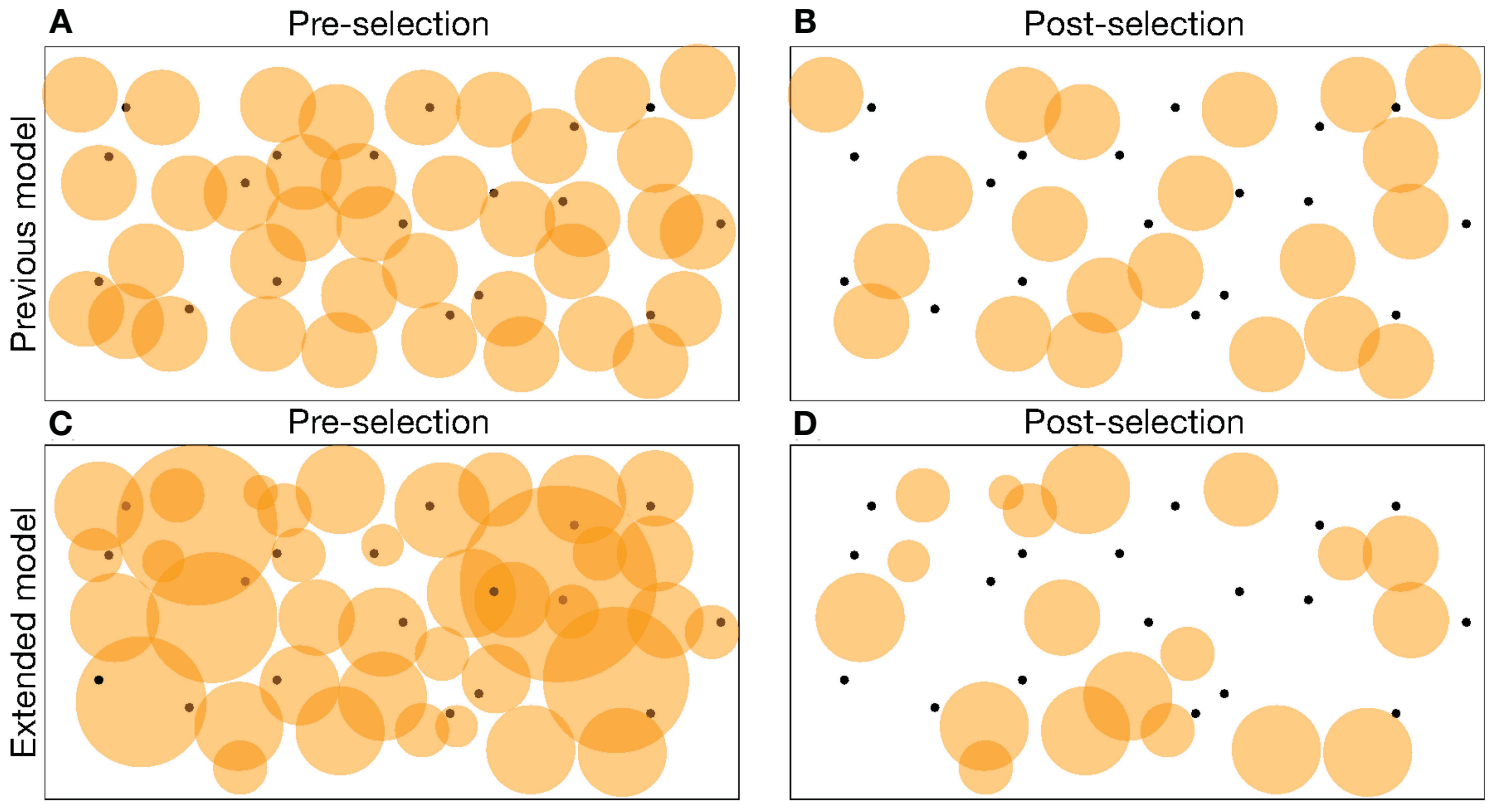
A cartoon of pre-selection **(A, C)** and post-selection **(B, D)** repertoires in a shape space representation. Clonotypes are depicted as orange circles representing the area in shape space that they cover. Self-epitopes are depicted as black dots. All clonotypes that cover at least one self-epitope have been deleted in the post-selection repertoires of **(B, D)**. In the previous model **(A, B)** all clonotypes have the same binding probability, *p*, making all circles equally large, whereas in the extended model **(C, D)** clonotypes differ in the degree of specificity, which is visualized as the size of their circles. In the extended model negative selection automatically selects for more specific clonotypes covering a smaller fraction of the shape space **(D)**.

The level of specificity at which a post-selection lymphocyte repertoire best responds to a foreign antigen was determined by analyzing simple mathematical models combining the probability of survival from negative selection with the probability to respond to a foreign epitope (14–16). The optimal binding probability was first shown to be inversely related to the number of self-epitopes the lymphocytes have to be tolerant to (15), and after allowing for incomplete tolerance this optimum was later confirmed to be an upper bound (14, 16). Thus, the typical precursor frequency of 1 in 10^5^ or 10^6^ clonotypes (i.e., cells expressing the same antigen receptors) (2–7) was thought to reflect an evolutionary adaptation of the lymphocyte specificity to not respond to about 10^5^ self-epitopes. In this work it was implicitly assumed that lymphocytes tend to have the same probability of binding pMHC, i.e., the same coverage of shape space (9); see **Figures 1A, B**.

Alternatively, experiments have suggested that the level of specificity of T-cell receptors in the postselection repertoire depends on the number of presented self-epitopes in the thymus (17–19). T cells obtained from mice expressing a single self-epitope in the thymus were found to be much more crossreactive than T cells obtained from normal mice (18). This suggests that T cells entering negative selection in the thymus have antigen receptors that differ markedly in their degree of specificity, and that clonotypes expressing crossreactive receptors are more likely to be removed from the repertoire when there are many self-epitopes (see **Figures 1C, D**). As a consequence, the level of specificity of the post-selection repertoire should be inversely related to the number of self-epitopes, and one would not need to invoke evolutionary optimization to explain the quantitative agreement between the typical number of self-antigens and the typical T-cell precursor frequency.

Mathematical models representing self-pMHC and T-cell receptors as strings of digits or amino acids have confirmed that negative selection makes the post-selection repertoire more specific (20, 21). Chao et al. (20), using differences between digits to define antigenic distance, were the first to confirm that negative selection is expected to decrease the average crossreactivity of the post-selection repertoire. These results were subsequently extended by Kosmrlj et al. (21), who defined self-pMHC and TCRs as strings of amino acids, and explicitly considered differences between strongly interacting and weakly interacting amino acids (22). This allowed them to predict that the T cells surviving negative selection should be enriched in weakly interacting amino acids (21). This prediction was recently confirmed by studies comparing the amino acid frequencies in the CDR3 regions of conventional (Tconv) and regulatory (Treg) CD4^+^ T cells (23, 24). Tregs are CD4^+^ T cells that have adopted a tolerized fate, e.g., after binding self-antigen(s) in the thymus, and can down-regulate immune responses (‘functional’ naive CD4^+^ T cells are conventional, i.e., Tconv cells). Stadinski et al. (23) showed that the presence of —the more interactive— hydrophobic amino acids in the middle of the CDR3 region predisposes cells to a Treg phenotype. Lagattuta et al. (24) showed that the more ‘sticky’ hydrophobic amino acids are enriched in Treg cells, while negatively charged amino acids are enriched in Tconv cells. Hydrophobic amino acids are also enriched in the relatively crossreactive T-cell receptors obtained from mice expressing just a single self-peptide (19). Thus, there is strong experimental evidence that negative selection weeds out the most crossreactive T cells on the basis of the ‘stickiness’ of the amino acids in their CDR3 regions.

We here address the question how this ‘mechanistic’ selection in the thymus on the basis of amino acid properties affects the average binding probability, i.e., the specificity level, of T lymphocyte receptors. We investigated whether the decrease in the binding probability that is due to negative selection is sufficient to explain the typical precursor frequency of 1:10^5^, or whether evolutionary selection has contributed as well to the exquisite specificity of lymphocytes.

## 2 Results

### 2.1 Optimal specificity

We previously developed a simple mathematical model for the probability, *P_i_*, that an immune response to a foreign antigen is mounted from a functional repertoire of *R* antigen receptors (15, 16). In these models, *p* is the probability that an antigen receptor binds a pMHC with an avidity exceeding the threshold for a cell to become activated and mount an immune response. We call *p* the ‘binding probability’ and we will use ‘epitope’ to refer to a particular pMHC. Specific T-cell receptors have a low value of *p* and crossreactive TCRs have a high value of *p*. Because this probability, *p*, directly defines the ‘precursor frequency’ of clonotypes responding to a foreign epitope, we know that 10^−6^ ≤ *p* ≤ 10^−5^ would be a reasonable range (2–7). In the models, *R*_0_ is the diversity of the pre-selection repertoire, i.e., the total number of unique antigen receptors made by V(D)J recombination, and *S* is the number of self-epitopes that require tolerance by clonal deletion, anergy or the formation of Tregs. The diversity of the post-selection (or functional) repertoire, *R*, is then determined by the probability, *P_s_* (for *P*_survival_), that a clonotype fails to recognize all self-epitopes *S*,


(1) $$\begin{array}{cc} R=R_{0}P_{s} & \text{where},\;P_{s}=\;{\left(1\;-p\right)}^{S} \end{array}$$

According to the simplest model based upon complete self tolerance (15), the probability that a functional repertoire of *R* TCRs fails to respond to a foreign epitope is the probability that none of its clonotypes recognize the epitope, *P_e_* = (1 − *p*)*^R^*, where the *e* stands for ‘escape’. Expressing one minus this chance of escape, as the probability of mounting an immune response to a foreign epitope, we obtain


(2) $$P_{i}=1-P_{e}=1\;-{\left(1-p\right)}^{R}=1-{\left(1-p\right)}^{R_{0}P_{s}}$$

Since ${\left(1-x\right)}^{n}≃{\text{e}}^{-xn}$ when *x* is small, we can approximate *P_s_* and *P_i_* by


(3) $$\begin{array}{ccc} P_{s}≃{\text{e}}^{-pS} & \text{and} & P_{i}≃1-{\text{e}}^{-pR_{0}P_{s}} \end{array}$$

The value of *p* that maximizes *P_i_* is computed by taking the derivative *∂_p_P_i_* and solving *∂_p_P_i_* = 0. One finds that the maximum is at *p* = 1*/S* (15). Evolution is therefore expected to select for individuals with lymphocyte binding probabilities around *p* = 1*/S*. Because $S≃10^{5}$ (10), this prediction was strikingly confirmed by the 1:10^5^ estimates for the T-cell precursor frequency (15).

**Figure 2:**
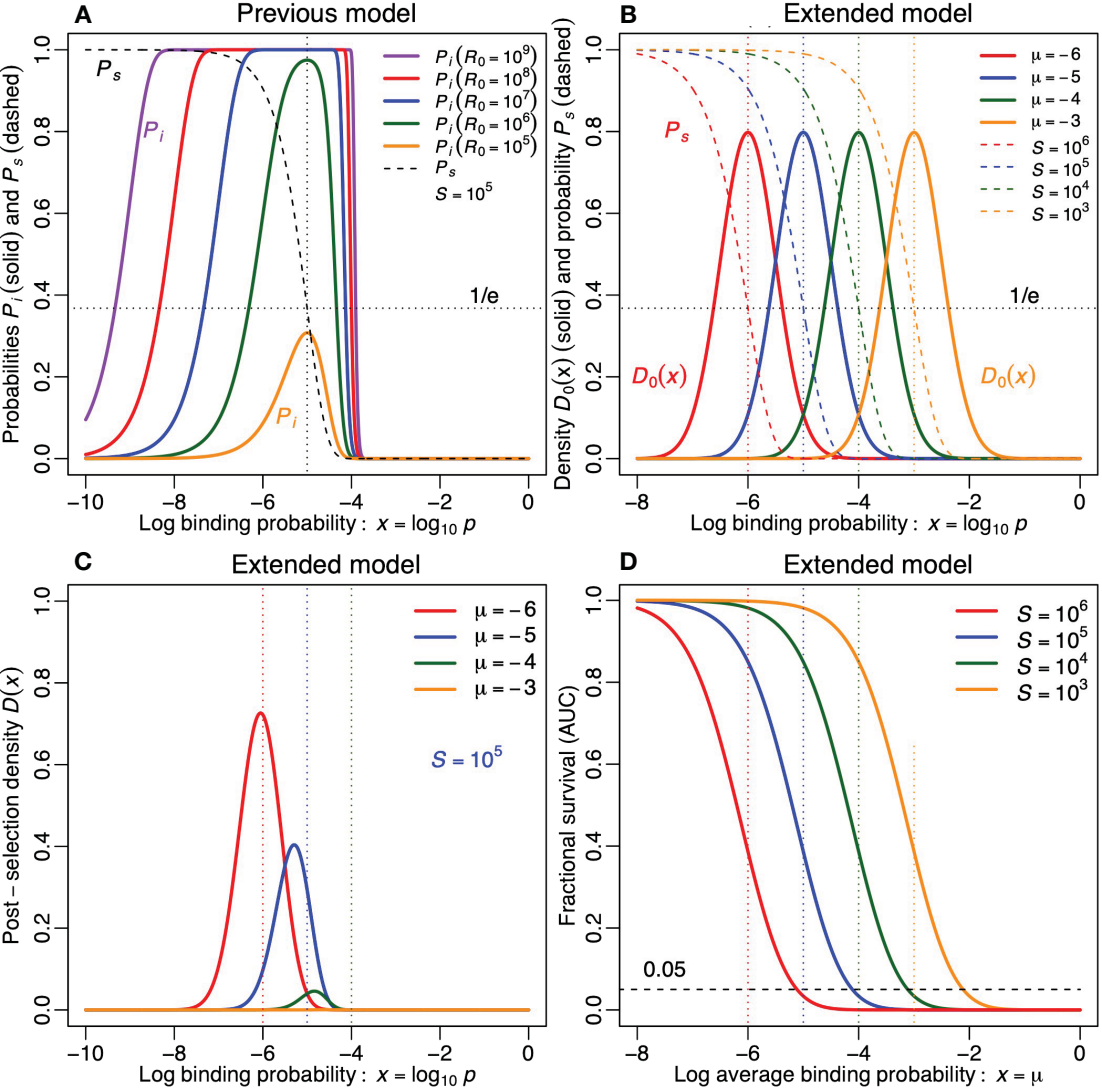
The impact of negative selection on the functional repertoire, in the previous **(A)** and in the extended model **(B–D)**. **(A)** The probability of mounting an immune response *P_i_* from Equation (2), and the probability of surviving tolerance induction *P_s_* from Equation (1), as a function of the log binding probability *p* of the lymphocytes (for 5 values of *R*_0_ and for *S* = 10^5^). The vertical dotted line denotes *p* = log_10_[1*/S*] = −5. The horizontal dotted line denotes *P_s_* = e^−1^. **(B)** The Gaussian functions depict the probability density function of the log binding probability of antigen receptors in the pre-selection repertoire, *D*_0_(*x*), for 4 values of *µ*. The declining sigmoid functions depict the probability of survival, *P_s_*, for 4 values of *S*. We match their color when *µ* = log_10_[1*/S*]. The vertical dotted lines depict the four values of *µ*. The horizontal dotted line denotes *P_s_* = e^−1^. **(C)** The distribution of the binding probability, *D*(*x*), of antigen receptors in the post-selection repertoire of Equation (5) for *S* = 10^5^. **(D)** The area under the curve of *D*(*x*), i.e., $\int_{-\infty }^{0}D(x)dx$ , as a function of *µ* for four values of *S*. The horizontal dashed line depicts that 5% of the double-positive thymocytes survives positive and negative selection. Note that the same survival is obtained when a 10-fold increase in *S* is perfectly compensated by a 10-fold decrease in *µ*. Parameters: *σ* = 0.5.

Taking the previously estimated *S* = 10^5^ self-epitopes (10) as an example, the probability of mounting an immune response, *P_i_*, is depicted in **Figure 2A** for pre-selection repertoires of *R*_0_ = 10^5^ to 10^9^ clonotypes (as the diversity of the pre-selection repertoire is expected to differ markedly between small and large animals). Large vertebrates like *Homo sapiens* have post-selection repertoires exceeding *R* = 10^9^ different T-cell clonotypes (1), and given that only 5% of the T cells maturing in the thymus survive positive and negative selection (25), should have pre-selection repertoires well exceeding *R*_0_ = 10^10^ different T-cell clonotypes. One of the smallest vertebrates is the fish species *Paedocypris* which is known to have about *R* = 37000 T cells (and about 12000 self-proteins) (26). Such a small species is not expected to be able to generate more than say *R*_0_ = 10^6^ different T-cell clonotypes. The *P_i_* curves in **Figure 2A** indeed have their optimum at *p* = 1*/S* = 10^−5^ (as indicated by the vertical dotted line). The dashed sigmoid in **Figure 2A** depicts the probability, *P_s_*, with which a clone with binding probability *p* survives negative selection, which illustrates that when *p* = 1*/S*, this probability becomes $P_{s}={\text{e}}^{-1}≃0.37$ (as indicated by the horizontal dotted line). Reassuringly, the predicted fraction of clonotypes surviving negative selection is higher than the estimated 5% T cells surviving both positive and negative selection (25).

In this simple model all cells were considered to have the same probability, *p*, of recognizing a random epitope. The models and the data reviewed in the Introduction suggest that the post-selection repertoire also becomes specific because thymic selection weeds out the most crossreactive clonotypes from the pre-selection repertoire (see **Figure 1D**). We therefore extend the model by allowing for a range of binding probabilities defined by a log normal distribution, with a mean *µ* and a standard deviation *σ*,


(4) $$D_{0}\left(x\right)=\frac{1}{\sigma \sqrt{2\pi }}\;{\text{e}}^{-{\left(x-\mu \right)}^{2}/\left(2\sigma^{2}\right)},$$

where *D*_0_(*x*) is a probability density, and *x* = log_10_ *p* defines a log specificity, meaning that *p* = 10*^x^*. Note that the log of the probability, *p*, obeys a normal distribution, that *p* = 1 when *x* = 0, that *D*_0_(*x*) is only defined for −∞< *x* ≤ 0, and that we are using a log_10_ for the specificity, instead of the conventional natural logarithm that is typical for a log normal distribution, because specificity levels are usually expressed as order of magnitudes (e.g., 10^−6^< *p<* 10^−5^). Since *D*_0_(*x*) is a probability density function it has an area under the curve of one. To define the total number of clonotypes, we therefore still need to multiply *D*_0_(*x*) with *R*_0_ (i.e., *R*_0_(*x*) = *R*_0_ *D*_0_(*x*)). The probability density function of Equation (4) is depicted in **Figure 2B** for various values of *µ* and for *σ* = 1*/*2. Note that for *σ* = 1*/*2 each repertoire contains a wide variation of antigen receptors, differing by several orders of magnitude in their specificity.

In the same panel of **Figure 2B** we also depict the survival probability, $P_{s}\left(x\right)={\text{e}}^{-pS}={\text{e}}^{-10^{x}S}$ from Equation (3) (see the dashed sigmoid lines representing *S* = 10^3^,10^4^, 10^5^ and 10^6^ self-epitopes). A 10-fold increase of *S* shifts the *P_s_* curve an order of magnitude to the left (as more clonotypes will be lost by tolerance induction).^1^ Since the solid *D*_0_(*x*) and the dashed *P_s_* (*x*) curves are independent, as the pre-selection repertoire *D*_0_ does not depend on *S*, it would still be possible to evolve an average specificity, *µ*, such that the *D*_0_(*x*) curve intersects the *P_s_* (*x*) curve at the same height in species with different numbers of self-epitopes, *S*. We therefore color both curves red, blue, green or orange, when log_10_[1*/S*] or *µ* equals −6,−5,−4 or −3, respectively (throughout the paper). This visualization reveals in **Figure 2B** that a similar level of survival during negative selection is expected when the average binding probability were decreased 10-fold for any 10-fold increase in *S*, which is similar to our previous results (14–16).

We study this further by explicitly defining the remaining density of receptors in the post-selection repertoire as *D*(*x*) = *P_s_* (*x*)*D*_0_(*x*),


(5) $$D\left(x\right)=\frac{1}{\sigma \sqrt{2\pi }}\;{\text{e}}^{-10^{x}S-{\left(x-\mu \right)}^{2}/\left(2\sigma^{2}\right)}\text{ and }R\left(x\right)=R_{0}D\left(x\right).$$

As an example, the probability density *D*(*x*) of the post-selection repertoire is depicted in **Figure 2C** for the previously estimated *S* = 10^5^ self-epitopes (10), and for various values of the mean, *µ*, of the lognormal distribution. Pre-selection repertoires composed of specific receptors, e.g., *µ* = −6, are hardly affected by tolerance induction to *S* = 10^5^ self-epitopes, whereas in repertoires composed of crossreactive receptors only a small fraction of the clonotypes survive tolerance induction to these *S* = 10^5^ self-epitopes (compare the height of the red *µ* = −6 curve with that of the green *µ* = −4 curve in **Figure 2C**, and compare the pre- and post-selection curves between **Figures 2B, C**). The fraction of clonotypes surviving tolerance induction can be quantified by plotting the area under the curve,^^2^^


(6) $$\text{AUC}=\frac{\int_{-\infty }^{0}R\left(x\right)dx}{R_{0}}=\int_{-\infty }^{0}D\left(x\right)\text{d}x,$$

as a function of the pre-selection average log specificity, *µ* (depicted for various values of *S* in **Figure 2D**). These curves reveal that whenever $p\gg 1/S(\text{or}\mu >\text{lo}{\text{g}}_{10}[1/S])$ , only a small fraction of the clonotypes survive tolerance induction. Since the combined survival of positive and negative selection was estimated to be 5% (25), the dashed horizontal line at *P_s_* = 0.05 depicts an experimental lower bound: negative selection by itself should not be lower than *P_s_* = 0.05. Together the curves in **Figure 2D** suggest that the average binding probability of the preselection repertoire cannot be larger than *µ* = −4 for *S* = 10^5^, *µ* = −3 for *S* = 10^4^ and *µ* = −2 for *S* = 10^3^; otherwise less than 5% of the clonotypes in the pre-selection repertoire survive negative selection. Because specific receptors preferentially survive, the post-selection distributions of the more crossreactive repertoires, e.g., *µ* ≥−5 (for *S* = 10^5^), are skewed to the left in **Figure 2C** (compare the location of the peaks with the color-matching vertical dotted lines at *µ* = −6,−5 and *µ* = −4). Thus, this model confirms that negative selection makes a crossreactive pre-selection repertoire more specific (18, 21).

**Figure 2D** confirms that one obtains the same fraction of clonotypes surviving (the same AUC), whenever a 10-fold increase in *S* is compensated by a 10-fold decrease in the average pre-selection binding probability, *µ*. This was already suggested by the color-matching curves in **Figure 2B**. Since the same fraction of clonotypes survive when an increase in *S* is perfectly compensated for by a decrease in the average binding probability, *µ*, the results remain similar to the optimum, *p* = 1*/S*, obtained with our earlier model (15), which did not consider a distribution of receptor binding probabilities. According to both models, species with more self-epitopes should thus have a more specific pre-selection repertoire to achieve a similar completeness of the functional post-selection repertoire.

### 2.2 Mounting immune responses

Because negative selection skews the binding probabilities, we explicitly compute the average binding probability of the post-selection repertoire of conventional T cells (by using the general definition of an average),


(7) $$\mu_{\text{Tconv}}=\frac{\int_{-\infty }^{0}xD\left(x\right)\text{d}x}{\int_{-\infty }^{0}D\left(x\right)\text{d}x}=\frac{\int_{-\infty }^{0}xD\left(x\right)\text{d}x}{AUC}\;.$$

**Figure 3:**
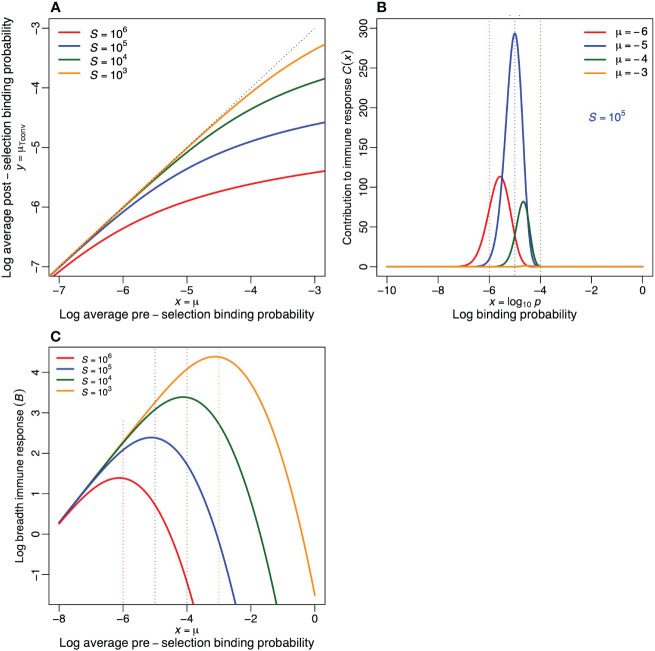
Properties of the functional post-selection repertoire in the extended model. **(A)** The average log binding probability, *µ*_Tconv_, of the post-selection repertoire defined by Equation (7). The dotted line depicts the diagonal (i.e., the situation where negative selection has no effect on the specificity of conventional T cells). This reveals that Tconv cells become more specific when there are more self-epitopes, and that this effect (i.e., the distance to the diagonal) increases when the pre-selection repertoire is more crossreactive. **(B)** The contribution to the immune response, *C*(*x*) = *R*_0_ *pD*(*x*), as defined by Equation (8), to a foreign antigen as a function of the log specificity, *x* (for *R*_0_ = 10^8^ and *S* = 10^5^). **(C)** The breadth of the immune response to a foreign antigen, $B=\int_{-\infty }^{0}C(x)dx$ , as a function of the average log specificity of the pre-selection repertoire, for four values of *S*. Parameters: *σ* = 0.5.

**Figure 3A** reveals that the average post-selection binding probability, *µ*_Tconv_, is always lower than the preselection binding probability, *µ* (observe that all curves are located below the diagonal). Moreover, the skewing of *µ*_Tconv_ increases when there are more self-epitopes, and when the pre-selection repertoire is more crossreactive (observe that the distance to the diagonal increases with *µ* and *S*). Despite this skewing, *µ*_Tconv_ increases monotonically with the average binding probability, *µ*, of the pre-selection repertoire.

Although the loss through negative selection increases with the crossreactivity of the pre-selection repertoire (**Figure 2D**), the Tconv clonotypes surviving selection in a crossreactive pre-selection repertoire, do have a relatively high probability to respond to a foreign antigen (**Figures 2C**, **3A**). To quantify the immune response we computed the expected breadth of an immune response. For functional clonotypes with a binding probability *p* = 10*^x^*, we define the contribution, *C*(*x*), to the immune response to a foreign epitope as *C*(*x*) = *pR*(*x*) = *pR*_0_ *D*(*x*),


(8) $$C\left(x\right)=\frac{R_{0}}{\sigma \sqrt{2\pi }}\;10^{x}{\text{e}}^{-10^{x}S-{\left(x-\mu \right)}^{2}/\left(2\sigma^{2}\right)},$$

which is depicted in **Figure 3B** for each specificity, *x*, and for various values of *µ*. This reveals that for *S* = 10^5^, the largest contribution is expected from cells with a binding probability of $p≃10^{-5}=1/S$ . The total number of clonotypes in an immune response, i.e., the breadth of the response, is then defined as the integral $B=\int_{-\infty }^{0}C(x)\text{d}x$ , which is depicted in **Figure 3C** for various values of *S*. This confirms that a pre-selection repertoire centered around *µ* = −5, is expected to mount the most diverse immune response to a foreign antigen (compare the location of the peaks with the color-matching vertical dotted lines). Note that this breadth, *B*, replaces the probability of an immune response, *P_i_*, of the previous model. Because due to its continuous nature there is always an immune response in the extended model (although it can become extremely narrow).

These results suggest that the binding probabilities of the functional post-selection repertoire are indeed determined by negative selection because the most crossreactive clonotypes in a pre-selection repertoire have the highest chance of becoming deleted. In **Figures 2C**, **3A** we saw that in crossreactive preselection repertoires, negative selection skews the distribution of binding probabilities to more specific clonotypes. Hence the previous observation (14–16) that the evolutionary optimum, *p* = 1*/S*, coincides with the typical precursor frequency (2–7), can also be explained by negative selection. Such a specific binding probability of the post-selection repertoire naturally follows from strong negative selection within a crossreactive pre-selection repertoire with many self-epitopes, e.g., for *µ* = −3 and *S* = 10^5^, see **Figure 3A**. Nevertheless, our extended model also confirms the previous results, as the optimal binding probability of the pre-selection repertoire remains to be centered around *p* = 1*/S* (see the color-matching vertical dotted lines in **Figure 3C**), because pre-selection repertoires composed of too specific receptors have a low probability to respond to foreign epitopes (**Figure 3B**), whereas repertoires composed of too crossreactive receptors suffer too much from clonal deletion (**Figure 2D**). Thus, evolution is still expected to select for immune systems with pre-selection lymphocyte binding probabilities centered around $p=1/S≃10^{-5}$ .

### 2.3 Optimizing the pre-selection repertoire

In addition to maximizing the probability of mounting an immune response by optimizing the recognition probability, we previously (15) also computed the size of the pre-selection repertoire required for having a sufficiently complete (8) functional repertoire for any given value of *p*. The probability that a foreign epitope is not recognized by any of the clonotypes in the functional repertoire was defined as *P_e_* = (1−*p*)*^R^* [see Equation (2) and (15)]. Solving *R*_0_ for a particular probability of escape, *P_e_*, corresponds to


(9) $$P_{e}=\left(1-p{)}^{R}≃{\text{e}}^{-pR}≃{\text{e}}^{-pR_{0}{\text{e}}^{-pS}}\text{ or }R_{0}≃-\text{ln }[P_{e}\right]\;\frac{{\text{e}}^{pS}}{p}\;.$$

**Figure 4:**
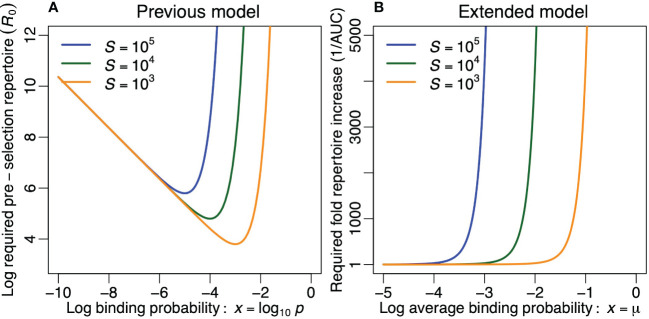
Optimizing the diversity of the pre-selection T-cell repertoire. **(A)** The required preselection repertoire diversity, *R*_0_, in the previous model, for *P_e_* = 0.1 per foreign epitope [see Equation (9)] and for three values of *S*. **(B)** The compensation required for keeping $\int_{-\infty }^{0}D(x)dx=1$ in the extended model for *σ* = 0.5 and for three values of *S*. Note that **(B)** is the inverse of **Figure 2D**.

Since most pathogens express several epitopes, picking *P_e_* ≤ 0.1 would allow most pathogens to be recognized.^3^ Plotting *R*_0_ as a function of *p* reveals that this function has a minimum (see **Figure 4A**), and solving *∂_p_R*_0_ = 0 shows that this minimum is again located at *p* = 1*/S*. At this minimum *R*_0_ = −ln[*P_e_*]e*S*, which is proportional to the number of self epitopes, *S*, and only depends logarithmically on the probability of escape, *P_e_*. This confirms that the immune system needs to be specific largely because there are so many self epitopes.^4^ For *p* values larger than 1*/S*, the required *R*_0_ rapidly becomes prohibitively large (see **Figure 4A**).

In the extended model a foreign epitope never completely escapes recognition, but the breadth of its immune response, *B*, can become extremely narrow. We can perform a similar analysis by increasing *R*_0_ to compensate for the loss of clonotypes due to negative selection. Thus, rescaling the area under the curve of the post-selection repertoire to one for every value of *µ*, we define *D_N_* (*x*) = *D*(*x*)*/*AUC, where the subscript *N* stands for ‘normalized’. The required compensation in the size of the pre-selection repertoire, 1/AUC, is depicted in **Figure 4B**. We again observe that this compensation becomes prohibitively large for repertoires that are considerably more crossreactive than *p* = 1*/S* (or *µ* = log_10_ 1*/S*). We conclude that both models agree on the fact that an unrealistically large pre-selection repertoire is required whenever the pre-selection repertoire is too crossreactive (**Figures 4A, B**). Evolution should therefore select for pre-selection binding probabilities in a medium range that does not exceed *p* = 1*/S* too much.

### 2.4 Regulatory T cells

In the extended model, negative selection selects for more specific receptors in the functional repertoire (**Figures 2C**, **3A**). The receptors that become negatively selected should therefore be less specific, i.e., more crossreactive. This prediction by Chao et al. (20) and Kosmrlj et al. (21) was recently confirmed by Lagattuta et al. (24), who demonstrated that the more ‘sticky’ hydrophobic amino acids, such as phenylalanine, leucine, tryptophan and tyrosine, are enriched in Treg cells, while the more weakly interacting amino acids, such as aspartic acid and glutamic acid, are enriched in Tconv cells. The repertoire of receptors that are negatively selected in our model is defined as


(10) $$D_{\text{Treg}}\left(x\right)=\left(1-P_{s}\left(x\right)\right)D_{0}\left(x\right).$$

**Figure 5:**
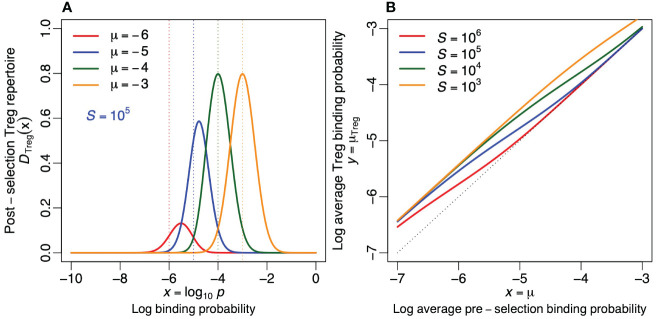
Regulatory T cells. **(A)** The probability density function of the post-selection Treg repertoire (as defined by Equation (10) for *S* = 10^5^). **(B)** The average log binding probability, *µ*_Treg_, of the post-selection Treg repertoires for four values of *S* [see Equation (11)]. The dotted line depicts the diagonal (i.e., the situation where negative selection has no effect on the specificity). Treg cells tend to become more crossreactive by negative selection (i.e., all curves are located above the diagonal). Parameters: *σ* = 0.5.


Loosely calling this the ‘Treg’ repertoire, *D*_Treg_(*x*) is depicted in **Figure 5A** (for various values of *µ* and for *S* = 10^5^). The area under the curve, and the average log specificity, are defined as

(11) $${\text{AUC}}_{\text{Treg}}=\int_{-\infty }^{0}D_{\text{Treg}}\left(x\right)\text{d}x\text{ and }\mu_{\text{Treg}}=\frac{\int_{-\infty }^{0}xD_{\text{Treg}}\left(x\right)\text{d}x}{AUC_{\text{Treg}}}\;.$$

Negative selection increases the crossreactivity of Tregs, especially when there are few self-epitopes and when the pre-selection repertoire is specific (see **Figure 5B**), because the most crossreactive receptors have the highest probability [1 − *P_s_* (*x*)] to become a Treg. The average binding probability of the Treg repertoire is hardly affected when there are many self-epitopes and the pre-selection repertoire is crossreactive because most receptors then become tolerized.

## 3 Discussion

Extending a previous model (15, 16) by allowing for a range of binding probabilities of T lymphocytes, we have confirmed the very natural notion (20, 21) that negative selection in the thymus is biased towards crossreactive T cells (18, 19, 24). Nevertheless, we have seen that binding probabilities of the TCRs in the pre-selection repertoire of a particular species need to be adapted to the number of self-antigens in that species to prevent massive deletion by negative selection. Additionally, lymphocyte receptors should not be too specific, as the functional post-selection repertoire needs to be fairly complete (8), i.e., cover most of shape space (9), to provide good protection from foreign antigens. Thus both evolution and negative selection play an important role in the exquisite binding probabilities of T cells.

We have modeled the fact that TCRs differ in their pMHC binding probabilities, e.g., due to the hydrophobicity of the amino acids in their CDR3 region (19, 23, 24). Similar effects may also play a role for MHC molecules presenting short peptides to T cells, as the polymorphic part of the MHC that forms part of the pMHC-TCR interface can also be composed of weakly and strongly binding amino acids. A significant part of the variation between the TCRs of Tregs and Tconvs can be attributed to binding the MHC molecule rather than the peptide (24). Based upon their modeling, Chao et al. (20) predicted that T cells binding their selecting MHCs strongly are more likely to become negatively selected. Thus, MHC alleles having strongly binding amino acids in the MHC-TCR interface would select a smaller T-cell repertoire. In our model, this would correspond to increasing the average level of crossreactivety, *µ*, of the pre-selection repertoire. Additionally, depending on the amino acids in the peptide binding groove, some MHC molecules could bind more peptides than others. Kosmrlj et al. (27) argued that particular MHC alleles do bind fewer self peptides than others (which remains somewhat uncertain because little is known about the absolute binding threshold of different MHC alleles), and that these MHCs hence select for a larger and more crossreactive functional repertoire. This would at least partly compensate for the lower number of foreign epitopes expected to be presented by such selective MHC molecules. Since in our model a more restrictive binding groove would correspond to decreasing the number of self-pMHC, *S*, the effects of these potential differences in the fraction of peptides bound by different MHC molecules can be predicted by changing *S* in the model (see **Figure 3**).^5^ Summarizing, since MHC molecules are polymorphic and differ in their binding properties, they may each select for a unique level of diversity and average specificity of the pool of T-cells restricted to them.

A considerable fraction of the TCRs in human T-cell repertoires lack a D-segment, and such sequences are preferentially generated during fetal development (28). Because D-segments tend to code for ‘non-sticky’ amino acids, with a strong enrichment of glycine (28), this suggests that the very early pre-selection T-cell repertoire is enriched in crossreactive receptors. Additionally, abundant TCR*β* sequences in naive T cell samples of young individuals tend to have high generation probabilities, short CDR3s, and absence of N additions (29–32). These differences suggest that TCRs may indeed differ widely in their binding probabilities. It is tempting to speculate that this early enrichment in crossreactive TCRs enables a rapid early coverage of the space of potential foreign antigens. However, our modeling also reveals that these receptors should not be too crossreactive, since they also need to survive negative selection. Nevertheless, it would seem beneficial to first fill the space with those crossreactive receptors that happen to survive negative selection, and later fill in the holes by making the pre-selection repertoire more specific.

Although there is promiscuous expression of self-antigens in the thymus (33), it remains unlikely that self tolerance is complete. Healthy individuals do harbor T cells that can recognize self-epitopes (3, 34, 35). Previously we have included potentially auto-reactive clonotypes in the model of Equation (2) by allowing a fraction of the self-pMHC to not impose negative selection (14, 16). A successful immune response was then defined as the probability of having an immune response from clonotypes not binding any of these ‘ignored’ self-epitopes. Since in species with large pre-selection repertoires, *R*_0_, the probability of mounting an immune response to a foreign epitope, *P_i_*, is close to one for a wide range of binding probabilities (see **Figure 2A**), it then becomes beneficial to have pre-selection binding probabilities lower than *p* = 1*/S* to reduce the probability of also recruiting potentially auto-reactive clonotypes into the immune response (14, 16). Hence the optimum *p* = 1*/S* of Equation (2) should be regarded as an upper bound, and *S* should be regarded as the number of self-epitopes imposing tolerance in the T-cell repertoire. Similarly, if not all self pMHC impose negative selection because some are ignored, have too low expression levels, or invoke indirect tolerance mechanisms, our estimate of *S* = 10^5^ (10) would be an upper bound. In most of our analyses we have therefore also considered 10 to 100-fold lower values of *S*. However, if some of the MHC molecules present more than 1% of the peptides, and/or if alternative splicing would allow for more than the predicted 10^7^ 9-mers in the human genome (10), one could also argue that *S* could be larger than 10^5^. Fortunately, we obtain qualitatively similar results for all values of *S*, i.e., all models agree that the pre-selection binding probability should not exceed p≃1/S .

In summary, our analyses confirm that the exquisite binding probability of functional T-lymphocytes in the circulation is naturally explained by negative selection on a large diversity of self-antigens. Nevertheless, evolution must have molded the binding probability of the pre-selection repertoire into a range that is compatible with the large diversity of self-antigens that are present in vertebrates, otherwise the post-selection repertoire would be too specific or too empty to respond to foreign intruders.

## Data availability statement

The original contributions presented in the study are included in the article. Further inquiries can be directed to the corresponding author.

## Author contributions

RB: Conceptualization, Writing – original draft, Writing – review & editing. CK: Conceptualization, Writing – review & editing. AP: Conceptualization, Writing – review & editing. JB: Conceptualization, Writing – review & editing.

## Footnotes

1 Because *P_s_* = e^−1^ when *p* = 1/*S*, the different *P_s_(x)* curves all approach *P_s_* ≃ 0.37 when *x* = log_10_1/*S*. See their intersections with the color-matching vertical dotted lines.

2 Since the maximum binding probability, *p* = 10*^x^* = 1, occurs when *x* = 0, the upper limit of the integral in Equation (6) is set to zero.

3 If all 37000 T cells in the very small fish *Paedocypris* (26) would be unique clonotypes, the probability that a foreign epitope is not recognized would even be larger, *P_e_* ≃e^−10−537000^≃0.7.

4 Note that *P*_*e*_≃1/e when *R* = 1/*p*, i.e., when one expects one response per epitope. The pre-selection repertoire *R*_0_ should then be 2.73-fold larger than the number of self-epitopes.

5 The probability to respond to a foreign epitope, *P_i_*, would not change, but a pathogen would be represented by fewer epitopes on MHCs with a more restrictive binding groove.
